# Human Chagas-Flow ATE-IgG1 for advanced universal and *Trypanosoma cruzi* Discrete Typing Units-specific serodiagnosis of Chagas disease

**DOI:** 10.1038/s41598-020-69921-z

**Published:** 2020-08-06

**Authors:** Glaucia Diniz Alessio, Fernanda Fortes de Araújo, Jéssica Spínola Silva, Policarpo Ademar Sales Júnior, Matheus de Souza Gomes, Laurence Rodrigues do Amaral, Juan David Ramírez, Carolina Flórez, Andréa Teixeira-Carvalho, Melina de Barros Pinheiro, Marta de Lana, Olindo Assis Martins-Filho

**Affiliations:** 1 Grupo Integrado de Pesquisas em Biomarcadores, Instituto René Rachou (FIOCRUZ-Minas), Belo Horizonte, MG Brazil; 2 Laboratório CT-Infra II, Histopatologia e Análises Clínicas, Universidade Federal de São João Del-Rei, Campus Centro Oeste Dona Lindu, Divinópolis, MG Brazil; 3 Grupo de Genômica Funcional e Proteômica de Leishmania spp e Trypanosoma cruzi, Instituto René Rachou (FIOCRUZ-Minas), Belo Horizonte, MG Brazil; 4 Laboratório de Bioinformática e Análises Moleculares, Rede Multidisciplinar de Pesquisa, Ciência e Tecnologia, Universidade Federal de Uberlândia, Campus Patos de Minas, Patos de Minas, MG Brazil; 5 Grupo de Investigaciones Microbiológicas-UR (GIMUR), Departamento de Biología, Facultad de Ciencias Naturales, Universidad del Rosario, Bogotá, Colombia; 6 Instituto Nacional de Salud, Bogotá, Colombia; 7 Laboratório de Doença de Chagas, Núcleo de Pesquisas em Ciências Biológicas (NUPEB), Instituto de Ciências Exatas e Biológicas (ICEB), Universidade Federal de Ouro Preto (UFOP), Ouro Preto, MG Brazil

**Keywords:** Microbiology, Parasitology, Immunology, Applied immunology, Biotechnology, Applied immunology

## Abstract

The molecular and serological methods available for Discrete Typing Units (DTU)-specific diagnosis of *Trypanosoma cruzi* in chronic Chagas disease present limitations. The study evaluated the performance of Human Chagas-Flow ATE-IgG1 for universal and DTU-specific diagnosis of Chagas disease. A total of 102 sera from Chagas disease patients (CH) chronically infected with TcI, TcVI or TcII DTUs were tested for IgG1 reactivity to amastigote/(A), trypomastigote/(T) and epimastigote/(E) antigens along the titration curve (1:250–1:32,000). The results demonstrated that “AI 250/40%”, “EVI 250/30%”, “AII 250/40%”, “TII 250/40%” and “EII 250/30%” have outstanding accuracy (100%) to segregate CH from non-infected controls. The attributes “TI 4,000/50%”, “EI 2,000/50%”, “AVI 8,000/60%” and “TVI 4,000/50%” were selected for DTU-specific serotyping of Chagas disease. The isolated use of “EI 2,000/50%” provided the highest co-positivity for TcI patients (91%). The combined decision tree algorithms using the pre-defined sets of attributes showed outstanding full accuracy (92% and 97%) to discriminate “TcI *vs* TcVI *vs* TcII” and “TcI *vs* TcII” prototypes, respectively. The elevated performance of Human Chagas-Flow ATE-IgG1 qualifies its use for universal and TcI/TcVI/TcII-specific diagnosis of Chagas disease. These findings further support the application of this method in epidemiological surveys, post-therapeutic monitoring and clinical outcome follow-ups for Chagas disease.

## Introduction

Chagas disease, caused by the parasite *Trypanosoma cruzi,* affects 8 million people worldwide mainly in Latin America^1^. The short-term acute phase of the disease evolves to long-lasting chronic phase with distinct clinical manifestations ranging from asymptomatic to cardiac, digestive or cardiac/digestive clinical forms^2–4^.

*Trypanosoma cruzi* presents a remarkable genetic diversity and has been classified into at least six Discrete Typing Units (DTUs) and an emerging DTU named TcBat^5^. Several studies have shown that besides selective geographical distribution of *T. cruzi* DTUs, the genetic variability is associated with distinct parasite biological behaviors, influencing the Chagas disease clinical outcome as well as the response to etiological treatment^6–15^. In this sense, the DTU-specific diagnosis of Chagas disease is a relevant approach not only for epidemiological surveillance underlying precise strategies for disease control but also as a reliable laboratorial tool for clinical prognosis and post therapeutic management^16,17^.

Molecular methods have been widely used for DTU-specific diagnosis of Chagas disease^5^. However, the use of these methods during chronic infection still represents a challenge. The requirement of parasite isolation by low sensitivity methods (hemoculture or xenodiagnosis) that may select *T. cruzi* genetic groups and the need of using several targets for a precise identification of distinct *T. cruzi* DTU are some of the major concerns^18–20^. Another limitation is that some molecular methods that require the parasite isolation do not obtain amplification due to the low number of copies of the mine-exon. Moreover, according to the clonal histiotropic model, the *T. cruzi* DTUs detected in peripheral blood samples do not necessarily represent those found at distinct host tissues^21–24^.

Aiming at overcoming these operational matters, innovative serological assays have been presented as promising devices for DTU-specific diagnosis of Chagas disease^25–28^. Regardless the considerable potential of the proposed ELISA-based serological methods to determine the *T. cruzi* infection repertoire, these methods showed to be not applicable to all lineage-specific serology for samples from distinct geographical regions. Battacharyya et al.^27^ demonstrated that TSSA lineage serology lacks specificity to detect TcI DTU. The association of *T. cruzi* peptides with others parasite antigens has been proposed as potential targets to improve the performance of ELISA-based serodiagnosis for Chagas disease^26^. However, the cross-reactivity of epitopes observed for hosts infected with distinct *T. cruzi* strains suggested that additional improvements are still required to achieve higher performance.

Recently, a flow cytometry-based test has been proposed as a strategy for DTU-specific serotyping. The Chagas-Flow ATE-IgG2a methodology has been standardized for the DTU-specific diagnosis of experimental *T. cruzi* infection displaying high performance to discriminate the hosts infected with distinct *T. cruzi* DTUs^29,30^. The present study show the Human Chagas-Flow ATE-IgG1 as a promising technique for advanced universal and DTU-specific serodiagnosis of Chagas disease.

## Methods

### Study population

This is an observational study that included a total of 102 patients with chronic Chagas disease (CH). The *T. cruzi* DTU isolated from each patient by hemoculture was identified for molecularly methods as previously described^18,19^. Based on the molecular data, the CH group was further categorized into three subgroups, according to the *T. cruzi* DTU infection, including: patients infected with TcI, from both genders, age > 18 years old, residents of Bogotá, Colombia (TcI infection, n = 35); patients infected with TcVI, from both genders, age > 18 years old, residents of Berilo, Jequitinhonha Valley, Minas Gerais, Brazil (TcVI infection, n = 07) and patients infected with TcII, from both genders, age > 18 years old, residents of Berilo (n = 45) and Bambui (n = 15), Minas Gerais, Brazil (TcII infection, n = 60). The control group of non-infected subjects comprised blood donors from both genders, age > 18 years old, residents of Belo Horizonte, Minas Gerais, Brazil (NI, n = 08). The serum samples were obtained from biorepositories maintained under responsibility of our group (JDR, ML and OAM-F). The genotyping profiles of those samples have been present elsewhere in original publications, including: TcII^31^, TcVI and TcII^20^ and TcI^32–34^. The serum samples from each participant was inactivated at 56 °C for 30 min and stored in aliquots at − 80 °C until use for Human Chagas-Flow ATE-IgG1 assay.

### Standard *T. cruzi* DTUs strains

In the present study, three standard *T. cruzi* strains were used as target antigens for Human Chagas-Flow ATE-IgG1, including: Colombiana strain, (TcI)^35^, CL strain (TcVI)^36^ and Y strain (TcII)^37^. The *T. cruzi* strains were obtained from the *T. cruzi* cryobank at Grupo de Genômica Funcional e Proteômica de *Leishmania spp* e *Trypanosoma cruzi*, Instituto René Rachou—FIOCRUZ-Minas and maintained in tissue and axenic cultures in vitro to prepare the amastigote (AMA-A), trypomastigote (TRYPO-T) and epimastigote (EPI-E) target antigens for Human Chagas-Flow ATE-IgG1.

### Preparation of target antigens for the Human Chagas-Flow ATE-IgG1

The amastigote, trypomastigote and epimastigote of the TcI, TcVI and TcII *T. cruzi* DTUs were obtained as described previously by Alessio et al.^30^. Briefly, live trypomastigotes (T) and amastigotes (A) forms were harvested from supernatant of desynchronized L929 cell line cultures in vitro after 4–6 days and 8–15 days post-infection, respectively. Live “A” & “T” suspension was maintained at 37 °C until fluorescent staining procedure.

Epimastigotes (E) forms were obtained at log-phase growth of axenic culture in vitro using “Liver Infusion Tryptose” (LIT) medium^38^. Pre-fixed “E” suspension was obtained by incubation overnight with FACS fix solution (10.0 g/L of paraformaldehyde, 10.2 g/L of sodium cacodylate and 6.65 g/L of sodium chloride, pH 7.2, all purchased from Sigma Aldrich, St Louis, MO, USA) diluted 1:2 in phosphate buffered saline PBS (0.15 M NaCl), pH 7.4, followed by washes in PBS and stored at 4ºC until fluorescent staining procedure.

Fluorescent staining procedures was carried out as described previously by Alessio et al.^39^. Briefly, live “A” & “T” antigens and pre-fixed “E” suspensions (1 × 10^7^ parasites/mL) were labeled with fluorescein isothiocyanate/FITC (Sigma Aldrich, St Louis, MO, USA) at 100 μg/mL for TcI and 200 μg/mL for TcVI and TcII, for 30 min at 37 °C. After staining, “A” & “T” suspension was re-incubated at 37 °C for 60 min for differential quenching of FITC labeling and the “E” suspension maintained at 4 °C overnight as described previously by Alessio et al.^39^. Prior using for Human Chagas-Flow ATE-IgG1, the fluorescent stained “A”, “T” and “E” mix of target antigens, displaying differential FITC label, were prepared and monitored by flow cytometry to certify the equivalent proportion of forms at final suspension (33% “A”, 33% “T” and 33% “E”).

### Human Chagas-Flow ATE-IgG1 assay

The Human Chagas-Flow ATE-IgG1 was carried out as described previously by Alessio et al.^30^, with some modifications. All serum samples were filtered (0.22 μm) to remove debris and submitted to serial dilution (1:250 to 1:32,000) in U-bottom 96-well plates. Aliquots of 50 μL were tested in parallel using each DTU-specific platform (TcI, TcVI and TcII target antigens) by incubation with of 50 μL of “A/T/E” mix of target antigens at 37 °C for 30 min. Following, parasites were washed twice with PBS supplemented with 10% fetal bovine serum (PBS-10%FBS), and re-incubated with 50 μL of pre-diluted (1:6,400) biotin-conjugated anti-human IgG1 (BD Bioscience, San Jose, CA, USA) plus 20 μL of pre-diluted (1:400) streptavidin phycoerytrin–SAPE (BD Bioscience, San Jose, CA, USA) at 37 °C for 30 min. “A/T/E” mix of target antigens were washed twice with PBS-10%FBS, fixed with 200 μL of FACS fixing solution and store at 4°C for 30 min and up to 24 h, prior to flow cytometer acquisition. Controls for second step reagents (anti-human IgG1-biotin + SAPE) were included in all experimental batches to monitor unspecific bindings.

Flow cytometric acquisitions were performed on a FACSCalibur flow cytometer (BD Bioscience, San Diego, CA, USA) using the CellQuest software Version 3.3 (URL https://www.bdbiosciences.com/documents/15_cellquest_prosoft_analysis.pdf) for data acquisition and storage. Instruments settings were applied on log scale (Forward Scatter-FSC = E00, Side Scatter-SSC = 427, threshold = 400; FL1 = 620 and FL2 = 500) and a total of 10,000 events were acquired per each tested serum dilution. Data analyses were performed using the FlowJo software Version 10.1 (TreeStar, San Diego, CA, USA, URL https://www.flowjo.com/solutions/flowjo/downloads). The IgG1 reactivity to “A/T/E” target antigens from each *T. cruzi* DTUs along the titration curve was expressed as percentage of positive fluorescent parasites (PPFP) according to Alessio et al.^30^, considering the shift of FL2 (α-IgG1-biotin/SAPE) outside the positivity limit (PPFP < 2%) established for the internal controls on one-dimensional histograms.

### Statistical and bioinformatics analysis

The GraphPad Prism software, Version 5.0 (San Diego, CA, USA, URL https://www.graphpad.com/scientific-software/prism/) was used for the descriptive statistical analysis. Comparative analysis of IgG1 reactivity to “A/T/E” target antigens from each *T. cruzi* DTU was carried out along the titration curve (1:250 to 1:32,000) to identify the pairs of attributes (“target antigens” & “serum dilution”) for universal and DTU-specific diagnosis for Chagas disease. Mann–Whitney test was employed for comparative analysis between CH and NI. Multiple comparisons amongst groups (TcI infection *vs* TcVI infection *vs* TcII infection) were carried out by Kruskal–Wallis test followed by the Dunns' post-test. In all cases, a threshold p value of < 0.05 was considered for statistical significance.

The R-project software, Version 3.0.1 (Vienna, Austria, URL https://www.R-project.org/) was used to calculate the modular distance between minimum and maximum IgG1 reactivity as well as between the median IgG1 reactivity along the titration curves (1:250 to 1:32,000) to further validate the pairs of attributes (“target antigen” & “serum dilution”) for universal and DTU-specific diagnosis of Chagas disease.

Receiver Operating Characteristic Curves (ROC) were constructed employing the MedCalc software, Version 7.3 (Ostend, Belgium, URL https://www.medcalc.org/) to define the cut-off edges and estimated the global accuracy based on the area under the ROC curve. Moreover, performance indices, expressed in percentage (sensitivity and specificity) were obtained for each set of attributes applied to the universal and TcI/TcVI/TcII-specific diagnosis of Chagas disease. The two-graph receiver operating characteristic (TG-ROC) curve was assembled to confirm the selected cut-off edges and identify the range of cut-off values (Δ = min and max ranges) with identical Se and Sp for each target antigen when applied for universal and TcI/TcVI/TcII-specific diagnosis purposes. The cut-off edges used for the TcI/TcVI/TcII-specific serotyping were calculated using the GraphPad Prism software, Version 5.0 (San Diego, CA, USA, URL https://www.graphpad.com/scientific-software/prism) employing the global median values for the selected sets of attributes.

The Minitab software, Version 18.1 (Pennsylvania, PA, USA, URL https://www.minitab.com/pt-br/downloads/) was used for principal component analyses (PCA) to further verify the ability of the selection pairs of attributes (“target antigen” & “serum dilution”) to segregate the Chagas disease patients infected with TcI, TcVI and TcII *T. cruzi* DTUs.

The WEKA software, Version 3.6.11 (Hamilton, New Zealand, URL https://www.cs.waikato.ac.nz/ml/weka/) was used to build the decision trees (root and branch) based on the pre-selected set of attributes (“target antigen”; “serum dilution” & “cut-off values”) and create algorithms for DTU-specific diagnosis of Chagas disease. The accuracy and the “leave-one-out-cross-validation” (LOOCV) values were used as performance scores.

The graphical arts (box plots, scatter charts, reactivity board panels and decision trees) were constructed using Microsoft Excel 2010 (URL https://www.microsoft.com/pt-br/microsoft-365/previous-versions/microsoft-excel-2010), PowerPoint 2010 software (URL https://www.microsoft.com/pt-br/microsoft-365/previous-versions/microsoft-powerpoint-2010) and GraphPad Prism software, Version 5.0 (San Diego, CA, USA, URL https://www.graphpad.com/scientific-software/prism/).

### Ethics statement

The present study was submitted and approved by the Ethical Committee at Instituto René Rachou—FIOCRUZ-Minas (C.A.A.E: 26890014.6.0000.5091, Protocol approval number # 3.055.734) and at Universidade Federal de Ouro Preto (C.A.A.E: 26890014.6.3001.5150, Protocol approval number # 766.573). All participants have read and sign the informed consent form.

## Results

### Overall reactivity of Human Chagas-Flow ATE-IgG1 for universal serodiagnosis of Chagas disease

**Figure 1:**
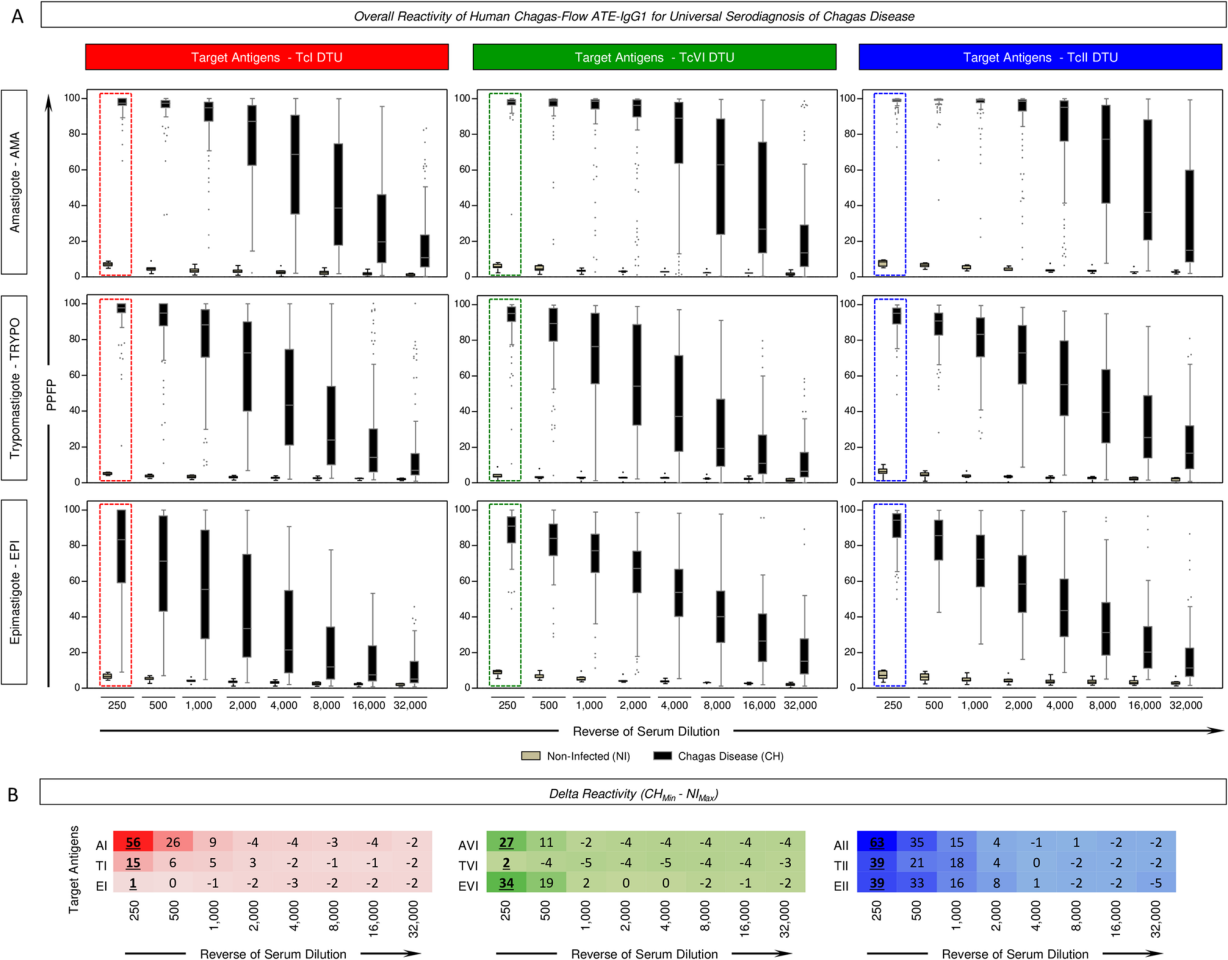
Overall reactivity of Human Chagas-Flow ATE-IgG1 for universal serodiagnosis of Chagas disease. Human Chagas-Flow ATE-IgG1 reactivity of serum samples from Chagas disease patients (black, n = 102) and non-infected controls (gray, n = 8). The IgG1 reactivity to each target antigen (amastigote-AMA, trypomastigote-TRYPO and epimastigote-EPI) from *T. cruzi* I (TcI, left panels), *T. cruzi* VI (TcVI, middle panels) and *T. cruzi* II (TcII, right panels) was assessed along the titration curve (1:250 to 1:32,000). (**A**) The results are expressed as the percentage of positive fluorescent parasites (PPFP) in box plot format, stretching from minimum to maximum values with outliers represented by dots and the box defining the interquartile range (25th and 75th) and the median value (line across the box). Comparative analyses between CH and NI at each serum dilution were carried out by the Mann–Whitney test. The dotted rectangles (red for TcI, green for TcVI and blue for TcII) underscore the pair of attributes (“target antigens” & “serum dilutions”) pre-selected as those with the higher segregation between CH and NI to be applied in the universal diagnosis of Chagas disease. (**B**) Delta reactivity of modular distance between minimum reactivity of CH group and maximum reactivity of NI individuals for each antigen and serum dilutions tested. The pairs of attributes “AI 250”, “TI 250”, “EI 250”, “AVI 250”, “TVI 250”, “EVI 250”, “AII 250”, “TII 250” and “EII 250” were selected as those with the higher delta reactivity (CH_Min_–NI_Max_) to be applied in the universal diagnosis of Chagas disease. Delta reactivity values with higher modular distance between minimum (CH_Min_) and maximum (NI_Max_) IgG1 reactivity for each antigen (AI, TI, EI, AVI, TVI, EVI, AII, TII and AII) are underscored by bold/underlined format.

The reactivity profiles of Human Chagas-Flow ATE-IgG1 for serum samples from Chagas disease (CH) patients and non-infected controls (NI) are shown in the Fig. 1.

The results are presented as median IgG1 reactivity along the titration curves (serum dilutions ranging from 1:250 to 1:32,000) for distinct target-antigens (amastigote-AMA, trypomastigote-TRYPO and epimastigote-EPI) from TcI (Fig. 1A, left panels), TcVI (Fig. 1A, middle panels) and TcII (Fig. 1A, right panels).

Comparative analysis of modular distance for IgG1reactivity between minimum of CH (CH_Min_) and maximum of NI (NI_Max_) allowed the pre-selection of the pair of attributes (“target antigens” & “serum dilutions”) with the higher delta reactivity (CH_Min_–NI_Max_) to be applied in the universal diagnosis of Chagas disease (Fig. 1B).

The results demonstrated that the pairs of attributes “AI 250”, “TI 250”, “EI 250”, “AVI 250”, “TVI 250”, “EVI 250”, “AII 250”, “TII 250” and “EII 250” presented the highest delta reactivity to segregated CH from NI (Fig. 1). These attributes were pre-selected for further assessment of performance applied to universal diagnosis purpose.

### Performance of Human Chagas-Flow ATE-IgG1 for universal serodiagnosis of Chagas disease

**Figure 2:**
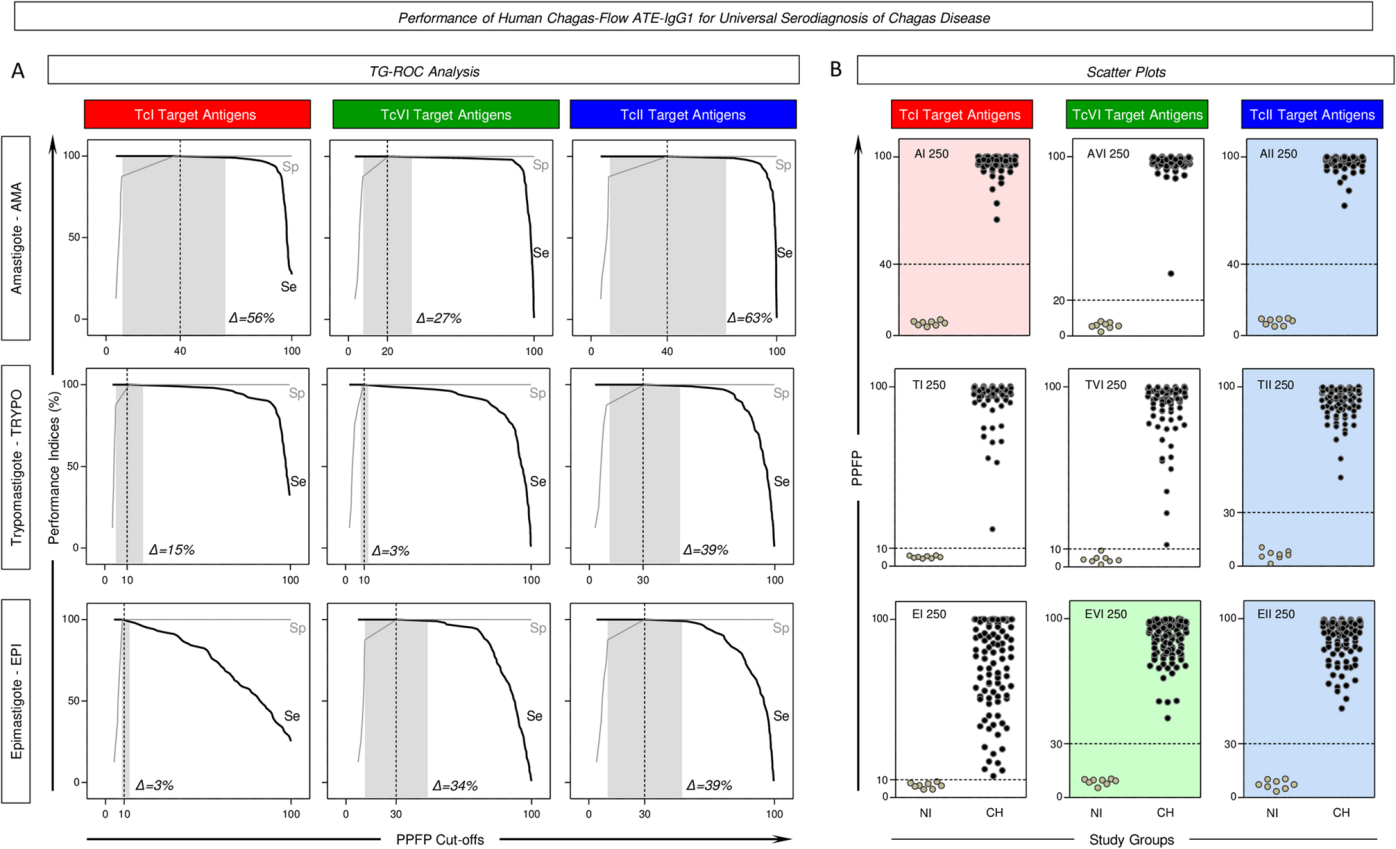
Performance of Human Chagas-Flow ATE-IgG1 for universal serodiagnosis of Chagas disease. (**A**) Two-graph receiver operating characteristic (TG-ROC) analysis of performance indices [Sensitivity (Se) and Specificity (Sp)] along a range of cut-offs for Human Chagas-Flow ATE-IgG1 applied to universal diagnosis of Chagas disease using distinct target antigens. The gray background underscored the range of cut-off values (Δ = min and max ranges) with identical Se and Sp for each target antigen. TG-ROC curve profiles pre-selected the pairs of attributes “AI 250”, “EVI 250”, “AII 250”, “TII 250” and “EII 250” as those with broader cut-off ranges to segregated sera samples from Chagas disease patients (CH) from non-infected controls (NI). (**B**) Representative scatter plot distributions of IgG1 reactivity at individual level further illustrate the ability of the pre-selected set of attributes to discriminate the IgG1 reactivity observed for CH (black dots) from NI (gray dots). The results are expressed as the percentage of positive fluorescent parasites (PPFP) with the dotted line representing the cut-offs defined by the TG-ROC analysis. Color backgrounds (red for TcI, green for TcVI and blue for TcII) highlight the sets of attributes (“target-antigen/serum dilution/cut-off”) with the higher performance for the universal diagnosis of Chagas disease, including “AI 250/40”, “EVI 250/30%”, “AII 250/40%”, “TII 250/40% and “EII 250/30%”.

The Fig. 2 displays the performance of the pre-selected pairs of attributes “AI 250”, “TI 250”, “EI 250”, “AVI 250”, “TVI 250”, “EVI 250”, “AII 250”, “TII 250” and “EII 250” for universal diagnosis of Chagas disease.

Two-graph receiver operating characteristic (TG-ROC) analysis were employed to define the most accurate cut-offs and define the min and max cut-off values (Δ = range) with identical performance indices for each pair of attributes (Fig. 2A). Using the proposed cut-offs, data analysis demonstrated excellent performance indices (Se = 100% and Sp = 100%) for all pre-selected attributes. However, detailed analysis of the TG-ROC curve profiles pointed out that the pair of attributes “AI 250”, “EVI 250”, “AII 250”, “TII 250” and “EII 250” presented broader cut-off ranges leading to identical performance indices (Δ = 56%, 34%, 63%, 39% and 39%, respectively) to segregated CH from NI (Fig. 2A).

Based on these findings, sets of attributes (“target antigen”, “serum dilution” & “cut-off values”) were defined for using in the universal diagnosis of Chagas disease, including: “AI 250/40%”, “EVI 250/30%”, “AII 250/40%”, “TII 250/40%” and “EII 250/30%” (Fig. 2A). Scatter plot distributions of IgG1 reactivity at individual level further illustrate these findings and confirm the outstanding performance of Human Chagas-Flow ATE-IgG1 for universal diagnosis of Chagas disease (Fig. 2B).

### Panoramic overview of Human Chagas-Flow ATE-IgG1 for DTU-specific serodiagnosis of Chagas disease

**Figure 3:**
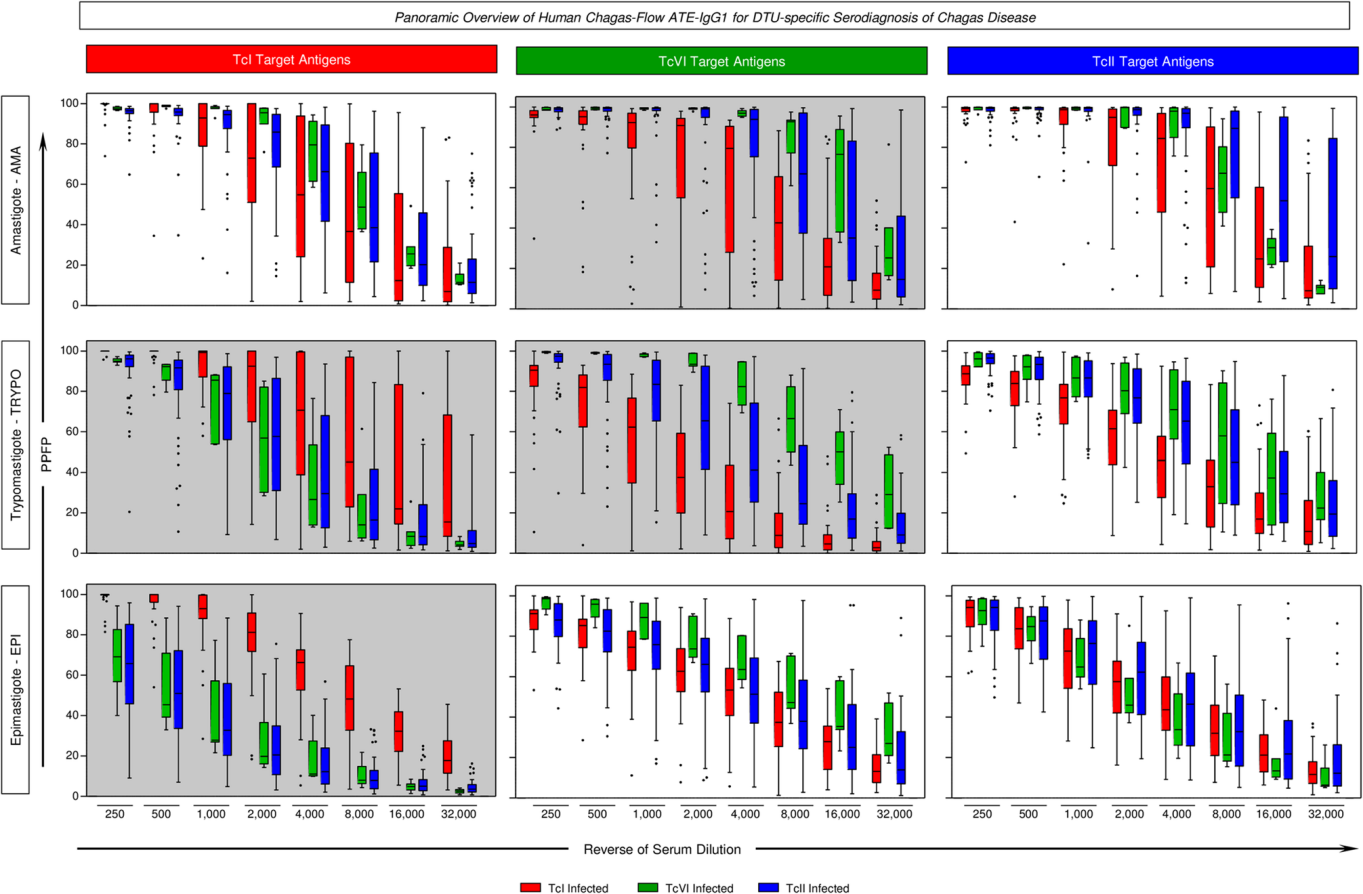
Panoramic overview of Human Chagas-Flow ATE-IgG1 for DTU-specific serodiagnosis of Chagas disease. Human Chagas-Flow ATE-IgG1 reactivity determined for serum samples from Chagas disease patients infected with TcI (red, n = 35), TcVI (green, n = 7) and TcII (blue, n = 60). The IgG1 reactivity to each target antigen (amastigote-AMA, trypomastigote-TRYPO and epimastigote-EPI) from *T. cruzi* I (TcI, left panels), *T. cruzi* VI (TcVI, middle panels) and *T. cruzi* II (TcII, right panels) was assessed along the titration curve (1:250 to 1:32,000). The results are expressed as the percentage of positive fluorescent parasites (PPFP) in box plot format, stretching from minimum to maximum values with outliers represented by dots and the box defining the interquartile range (25th and 75th) and the median value (line across the box). Comparative analyses amongst groups of CH patients (TcI vs TcVI vs TcII) at each serum dilution were performed by the Kruskal–Wallis followed by Dunn’s post test for multi-group comparisons. Gray background underscores the target antigens with higher ability to segregate the IgG1 reactivity amongst CH patients infected with TcI, TcVI and TcII. The target antigens “TI”, “EI”, “AVI” and “TVI” were pre-selected for DTU-specific diagnosis of Chagas disease as they present less overlapping of data distribution from lower to upper whisker.

The overall profiles of IgG1 reactivity of serum samples from patients infected with TcI, TcVI and TcII *T. cruzi* DTUs were assessed by Human Chagas-Flow ATE-IgG1 and the data are presented in the Fig. 3. The data are shown as median IgG1 reactivity along the titration curves (1:250 to 1:32,000) for distinct target antigens (AMA = “A”, TRYPO = “T” and EPI = E) of TcI (Fig. 3, left panels), TcVI (Fig. 3, middle panels) and TcII (Fig. 3, right panels).

Multiple comparisons amongst patients infected with distinct *T. cruzi* DTUs allowed the selection of target antigens with the most outstanding profile for using in DTU-specific serotyping of Chagas disease. Box plot analysis illustrate that “TI”, “EI”, “AVI” and “TVI” target antigens displayed less overlapping of data distribution from lower to upper whisker (Fig. 3). These target antigens were selected for further performance assessment to the DTU-specific diagnosis of Chagas disease.

### Pre-selection of the pair of attributes (“target antigens” and “serum dilution”) for DTU-specific serodiagnosis of Chagas disease

**Figure 4:**
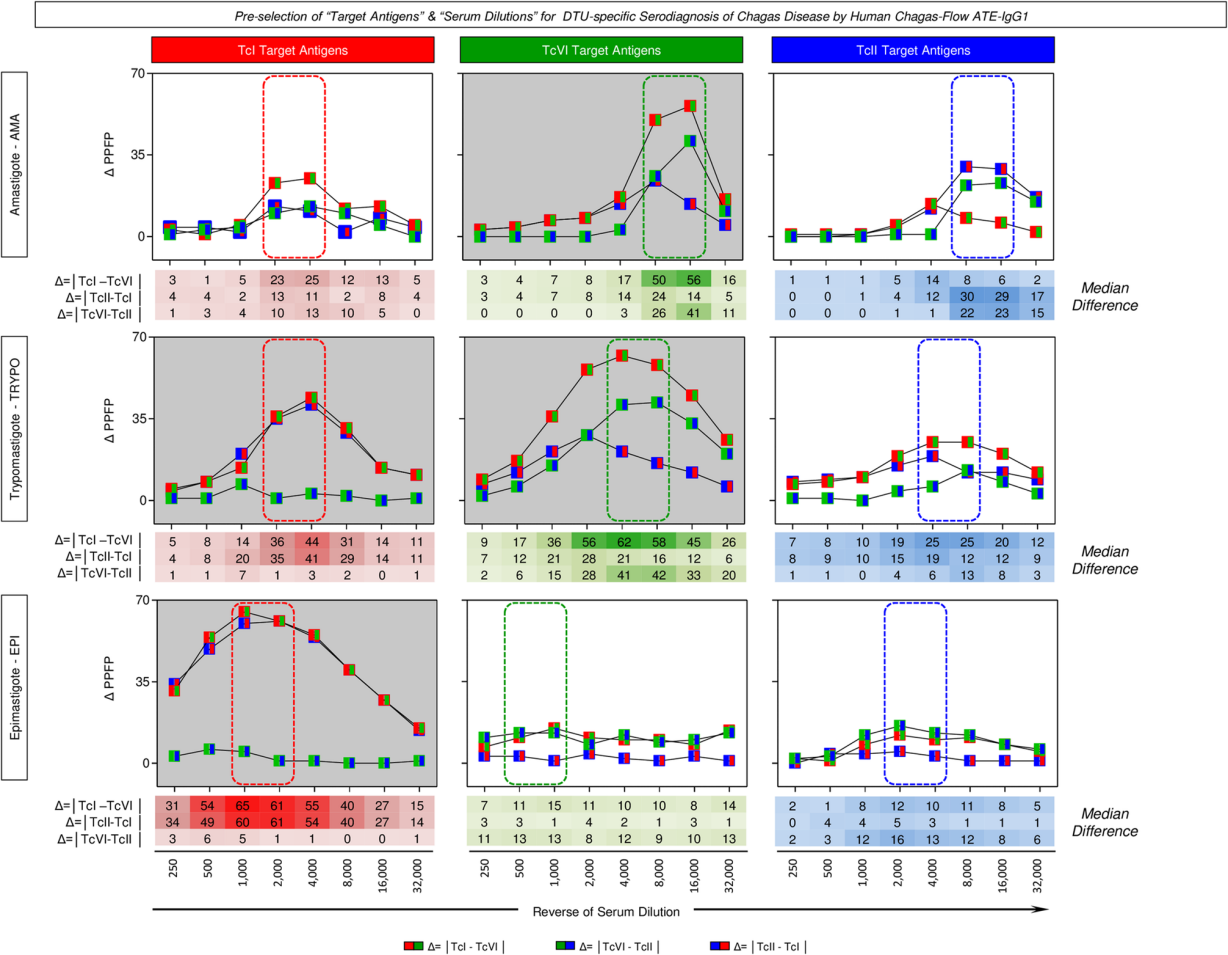
Pre-selection of target antigens and serum dilution for DTU-specific serodiagnosis of Chagas disease by Human Chagas-Flow ATE-IgG1. The Euclidean distance (Δ = │TcI − TcVI│, Δ = │TcII − TcI│ and Δ = │TcVI − TcII│) of median reactivity calculated along the titration curves (1:250 to 1:32,000) for serum samples from patients infected with TcI, TcVI and TcII *T. cruzi* DTUs. The data are presented as differential reactivity expressed as the percentage of positive fluorescent parasites (Δ PPFP). The dotted rectangles (red for TcI, green for TcVI and blue for TcII) underscore the pair of attributes (“target antigens” & “serum dilutions”) with higher delta reactivity to segregate subgroups of Chagas disease patients. Gray background highlights the pre-selected pairs of attributes (“TI 2,000, “TI 4,000), (“EI 1,000”, “EI 2,000”), (“AVI 8,000, “AVI 16,000”) and (“TVI 4,000”, “TVI 8,000”) for further performance evaluation applied to the DTU-specific diagnosis of Chagas disease.

The Euclidean distance (Δ = │TcI − TcVI│, Δ = │TcII − TcI│ and Δ = │TcVI − TcII│) of median reactivity were calculated along the titration curves (1:250 to 1:32,000) for serum samples from patients infected with TcI, TcVI and TcII *T. cruzi* DTUs and data are present in the Fig. 4.

The modular distance for each target antigen (AMA = “A”, TRYPO = “T” and EPI = E) of TcI (Fig. 4, left panels), TcVI (Fig. 4, middle panels) and TcII (Fig. 4, right panels) were then employed for pre-selecting the pair of attributes (“target antigens” & “serum dilutions”) with higher delta reactivity to segregate subgroups of Chagas disease patients. The results allowed the pre-selection of promising pair of attributes (“TI 2,000, “TI 4,000), (“EI 1,000”, “EI 2,000”), (“AVI 8,000, “AVI 16,000”) and (“TVI 4,000”, “TVI 8,000”) for further performance evaluation applied to the DTU-specific diagnosis of Chagas disease (Fig. 4).

### Final selection of set of attributes (“target antigen”, “serum dilution” & “cut-off values”) for DTU-specific serodiagnosis of Chagas disease

**Figure 5:**
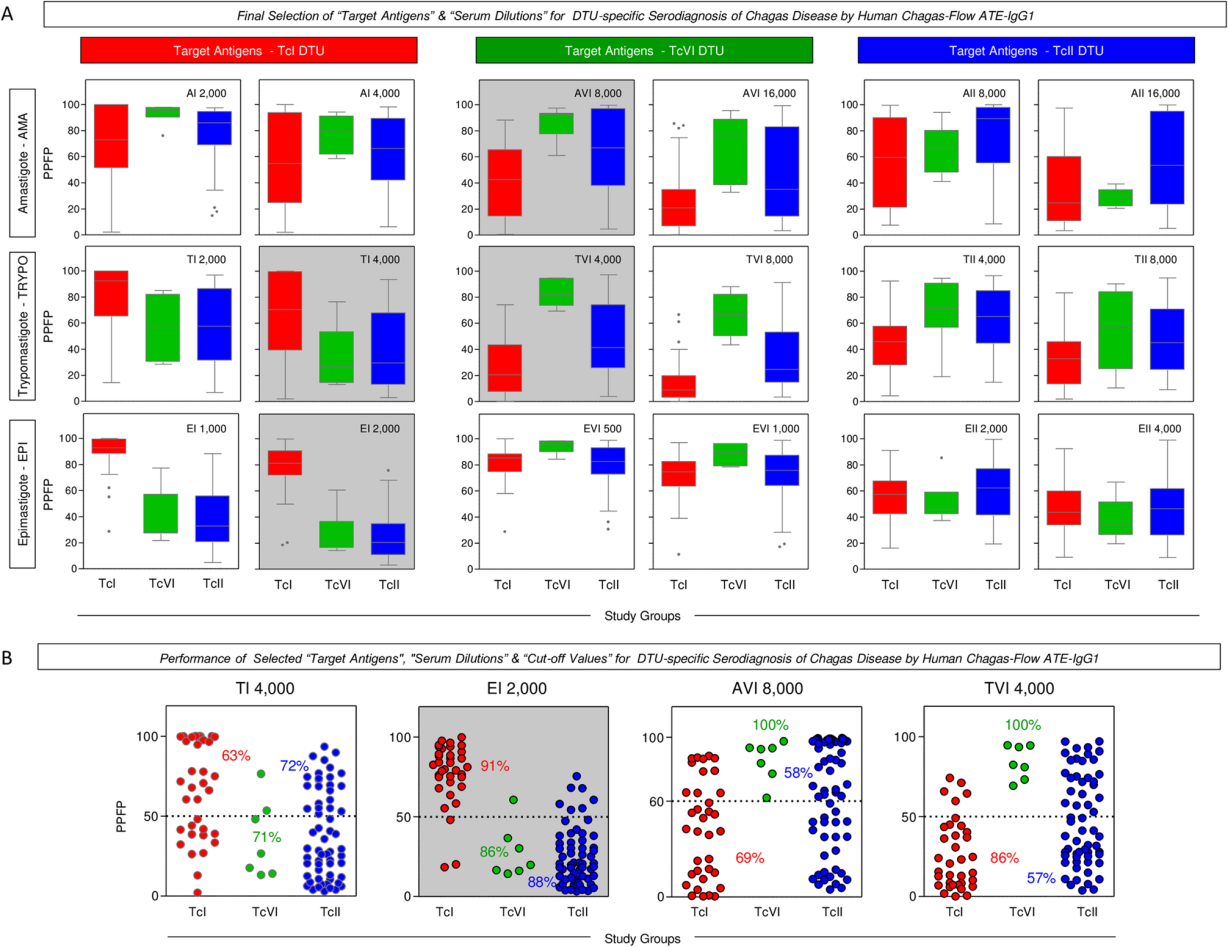
Final selection of target antigens and serum dilutions for DTU-specific serodiagnosis of Chagas disease by Human Chagas-Flow ATE-IgG1. (**A**) Multiple comparison of median IgG1 reactivity carried out to define sets of attributes with better performance to segregate the IgG1 reactivity observed for Chagas disease patients infected with TcI (red, n = 35), TcVI (green, n = 7) and TcII (blue, n = 60). The results are expressed as the percentage of positive fluorescent parasites (PPFP) in box plot format, stretching from minimum to maximum values with outliers represented by dots and the box defining the interquartile range (25th and 75th) and the median value (line across the box). Comparative analyses were performed by the Kruskal–Wallis followed by Dunn’s post test for multi-group comparisons. The pairs of attributes “TI 4,000”, “EI 2,000”, “AVI 8,000” and “TVI 4,000” were selected as the most outstanding for DTU-specific diagnosis, according to the lower extent of overlap of interquartile ranges amongst patients infected with TcI, TcVI and TcII *T. cruzi* DTUs. (**B**) Scatter plots distributions of selected set of attributes (“target-antigen/serum dilution/cut-off”) to discriminate the reactivity of serum samples amongst Chagas disease patients (TcI, TcVI and TcII). The results were expressed as the range of PPFP values in scatter plots for individual serum samples. The dotted line represents the cut-off for each target-antigen/serum dilution. Median values of PPFP range were used to define the cut-off edge to discriminate positive and negative results. Gray background highlighted the performance of “EI 2,000/50%” as the set of attributes with higher co-positivity for patients infected with TcI (91%) and co-negativity for patients infected with TcVI and TcII (86% and 88%, respectively).

Multiple comparison of median IgG1 reactivity were carried out to define sets of attributes for DTU-specific serodiagnosis of Chagas disease and the results are presented in Fig. 5.

Data analysis demonstrated that “TI 4,000”, “EI 2,000”, “AVI 8,000” and “TVI 4,000” were the most outstanding pairs of attributes for DTU-specific serotyping based on the lesser overlapping interquartile ranges amongst patients infected with TcI, TcVI and TcII *T. cruzi* DTUs (Fig. 5A).

The global median values of IgG1 reactivity were used to define the cut-off edges to segregate positive and negative results for each pre-selected pair of attributes. Based on these data, pairs of attributes were defined, including: “TI 4,000/50%”, “EI 2,000/50%”, “AVI 8,000/60%” and “TVI 4,000/50%” (Fig. 5B).

The defined sets of attributes displayed an overall moderate accuracy when used as an isolate parameter for DTU-specific diagnosis of Chagas disease. While “TI 4,000/50%” showed a general low performance for DTU-specific serotyping of Chagas disease, “AVI 8,000/60%” and “TVI 4,000/50%” displayed high co-positivity to diagnose the infection with TcVI. Of note was the performance of “EI 2,000/50%” showing high co-positivity = 91% for patients infected with TcI DTU and moderate co-negativity = 86% and 88% for patients infected with TcVI and TcII DTUs, respectively (Fig. 5B).

### Performance of combined Human Chagas-Flow ATE-IgG1 for DTU-specific diagnosis of Chagas disease

**Figure 6:**
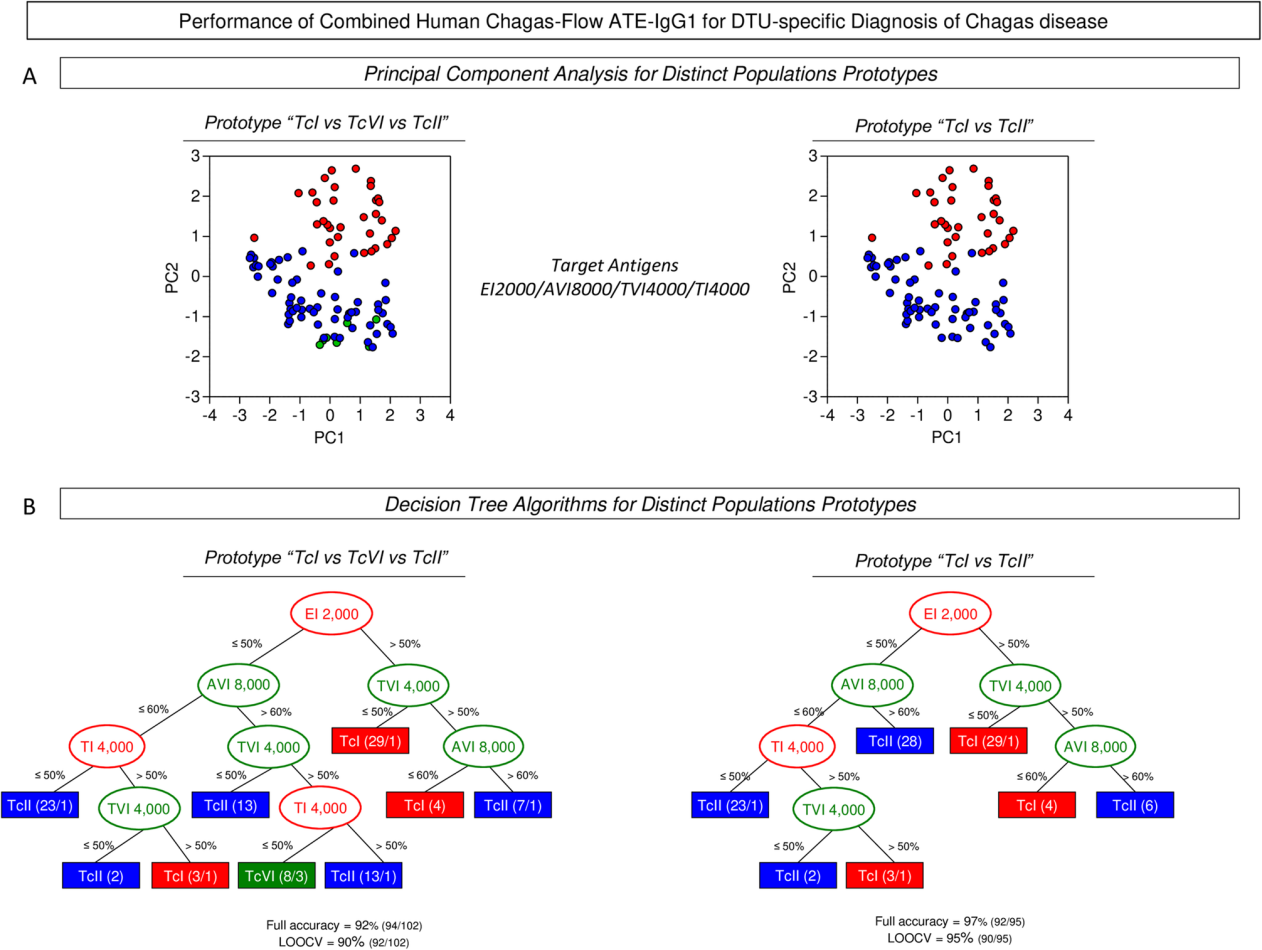
Performance of combined Human Chagas-Flow ATE-IgG1 for DTU-specific diagnosis of Chagas disease. (**A**) Principal component (PC) analysis of defined sets of attributes (“TI 4,000/50%”, “EI 2,000/50%”, “AVI 8,000/60%” and “TVI 4,000/50%”) were able to segregate sera samples from Chagas disease patients infected with distinct DTUs. Two population prototypes, referred as “TcI *vs* TcVI *vs* TcII” and “TcI *vs* TcII” were tested to illustrate the distribution of human *T. cruzi* infection in distinct geographic regions within the endemic area. Data are expressed as individual scores for PC1 and PC2. (**B**) Decision tree algorithms were built for DTU-specific diagnostic purposes for both population prototypes using the selected sets of attributes (“target-antigen/serum dilution/cut-off”). The global accuracy and leave-one-out-cross-validation (LOOCV) scores are provided in the Figure.

Aiming at improving the performance of Human Chagas-Flow ATE-IgG1 for DTU-specific diagnosis of Chagas disease, combined strategies for using the defined sets of attributes (“TI 4,000/50%”, “EI 2,000/50%”, “AVI 8,000/60%” and “TVI 4,000/50%”) were evaluated and the results presented in the Fig. 6. Two population prototypes, referred as “TcI *vs* TcVI *vs* TcII” and “TcI *vs* TcII” were tested to illustrate the distribution of human *T. cruzi* infection in distinct geographic regions around the world.

At first, principal component analyses were carried out to obtain a panoramic snapshot of using the combined sets of attributes (Fig. 6A). The results clearly demonstrated the ability of the target antigens to cluster the serum samples from TcI and TcII in both population prototypes (Fig. 6A). However, it was observed for the population prototype “TcI *vs* TcVI *vs* TcII” that TcVI serum samples clustered away from TcI but together with TcII serum samples (Fig. 6A).

Decision tree algorithms were built for DTU-specific diagnostic purposes for both population prototypes and the data showed in the Fig. 6B. In both cases, the “EI 2,000/50%” was indicated as root attribute followed by “AVI 8,000/60%”, “TVI 4,000/50%” and “TI 4,000/50%” as subsequent branch nodes. Data analysis showed high full accuracy and cross-validation for population prototype “TcI *vs* TcVI *vs* TcII” (Full = 92%, LOOCV = 90%) and remarkable performance indices for the population prototype “TcI *vs* TcII” (Full = 97%, LOOCV = 95%) (Fig. 6B).

### Overall performance of Human Chagas-Flow ATE-IgG1 for DTU-specific diagnosis of Chagas disease

**Figure 7:**
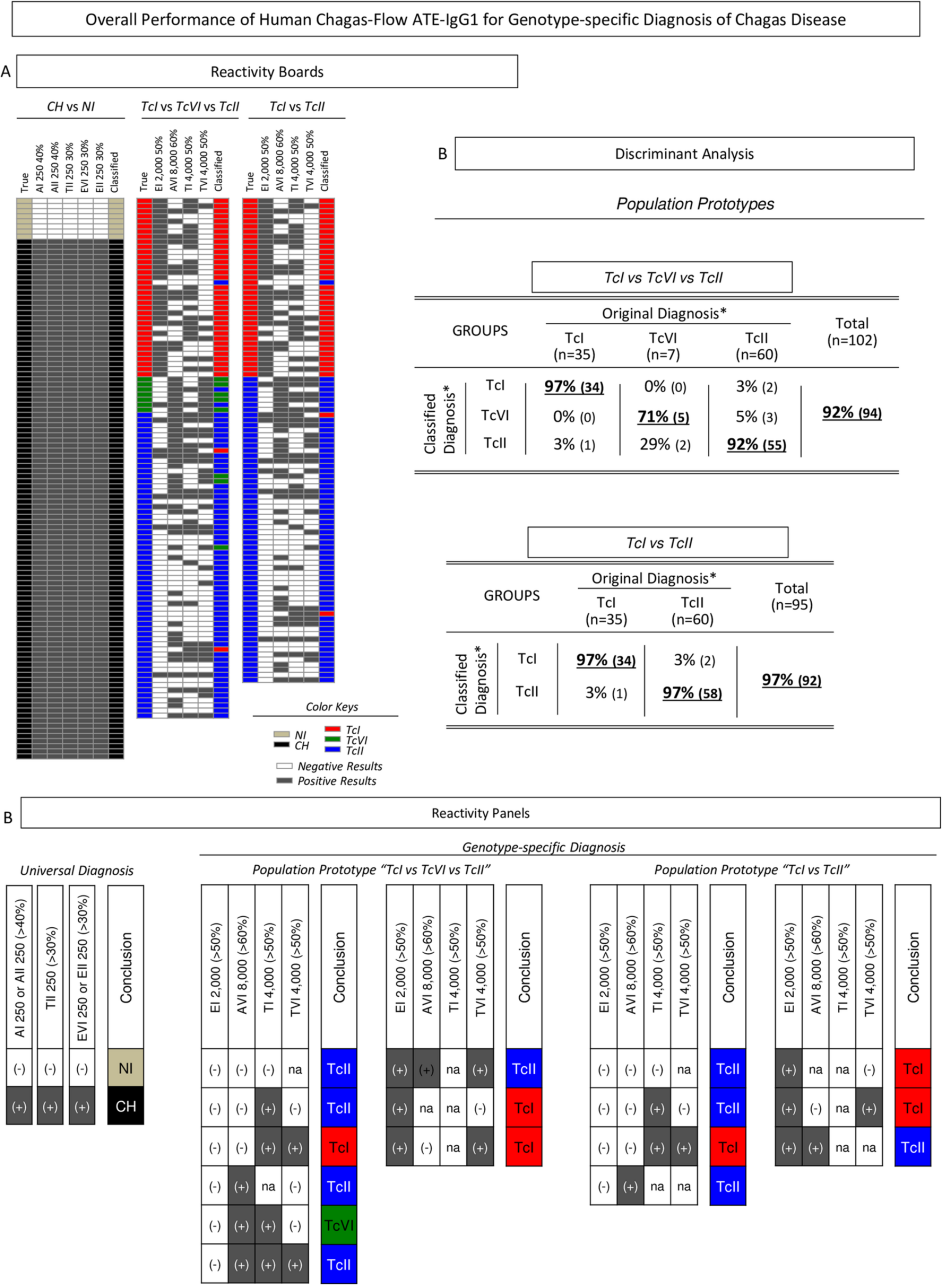
Overall performance of Human Chagas-Flow ATE-IgG1 for DTU-specific diagnosis of Chagas disease. (**A**) Reactivity boards of “Original Diagnosis” and “Classified Diagnosis” data for individual samples tested for universal (CH *vs* NI, left diagram) and DTU-specific diagnosis for two population prototypes TcI *vs* TcVI *vs* TcII (central diagram) and TcI *vs* TcII (right diagram). “Original Diagnosis” comprises the DTU identified by molecular approaches used as reference “gold standard” method to categorize the samples. “Classified Diagnosis” refers to the results achieved by serological analysis by Human Chagas-Flow ATE-IgG1. The selected sets of attributes (“target-antigen/serum dilution/cut-off”) determined by the decision tree analysis were applied in sequential algorithms to yield positive results (dark grey) and negative results (white) and define the “Classified Diagnosis” status to each individual samples using a color key panel to identify non-infected controls (NI, light brown), Chagas disease infected (CH, black), TcI infection (red), TcVI infection (green) and TcII infection (blue). (**B**) Discriminant analyses of combined Chagas-Flow ATE-IgG1 were employed to calculate the overall performance of Human Chagas-Flow ATE-IgG1 for DTU-specific diagnosis of Chagas disease. (**C**) Reactivity panels for selected sets of attributes compile all possibilities of sequential discretized data and define the diagnostic conclusions for universal and DTU-specific diagnosis of Chagas disease according to a color key panel: TcI (red), TcVI (green) and TcII (blue) and underscores the not applicable cases (n.a.).

The overall performance of Human Chagas-Flow ATE-IgG1 was estimated after data discretization using the selected sets of attributes for gathering the reactivity profile of individual samples (Fig. 7).

Reactivity boards were constructed for comparative analysis between “Original Diagnosis” and “Classified Diagnosis” data for individual samples tested for universal (CH *vs* NI) and DTU-specific diagnosis (Fig. 7A). “Original Diagnosis” comprises the DTU identified by molecular approaches used as reference “gold standard” method to categorize the samples. “Classified Diagnosis” refers to the results achieved by serological analysis by Human Chagas-Flow ATE-IgG1. The data was then employed to perform discriminant analysis and calculate the overall performance of Human Chagas-Flow ATE-IgG1 for DTU-specific serotyping of Chagas disease. The results demonstrated high accuracy of Human Chagas-Flow ATE-IgG1 for both population prototypes (92% and 97%, respectively) (Fig. 7B).

Reactivity panels for selected sets of attributes were assembled to compile all possibilities of sequential discretized data and define the diagnostic conclusions for universal and DTU-specific diagnosis of Chagas disease (Fig. 7C).

## Discussion

The distinct geographic distribution of *T. cruzi* DTUs in Latin America is well known and shows a clear predominance of TcII, TcV and TcVI in the domestic cycle as well as TcI in the sylvatic and domestic cycle of Chagas disease^5,15,40–45^. Besides influence in the epidemiological surveillance and control of Chagas disease according to the geographical region^17^, the diversity of *T. cruzi* DTUs is also associated with distinct disease severity and therapeutic response in *T. cruzi* infection^8,9,46–49^. Moreover, the genetic variability of *T. cruzi* can also interfere in the performance of the serological methods used for Chagas disease diagnosis^50–52^. Padilla et al.^53^ presented a new and complete target product profile (TPP) for the development of tests for the diagnosis and early assessment of Chagas disease treatment efficacy in order to assist the management of patients and control of the disease. The tests used for the diagnosis of Chagas disease should be universal, i.e., capable of detecting all human-infecting lineages^53^. In this sense, the development and standardization of laboratorial methods for accurate identification of distinct *T. cruzi* DTUs are relevant not only for epidemiological studies but also for a realistic application for patient management in the clinical practice.

Several available methods for DTU-specific diagnosis of *T. cruzi* infection are based on molecular biology techniques^18,19^. Although these molecular approaches are undoubtedly useful diagnostic tools for Chagas disease, some drawbacks should be considered, especially when applied for diagnosis of chronic infection. Besides requiring a combination of several genetic markers to detect and distinguish *T. cruzi* DTUs^18–20,54^, the need of previous parasite isolation may lead to clonal selection that not necessarily represent all genetic groups of *T. cruzi* infecting the host^19,21–24,55–57^. In addition, when molecular methods (PCR) are applied directly on the blood the sensitivity is low since some selected markers presented relative small number of copies in the *T. cruzi* genome^32,34^.

Several serological methods have already been developed to overcome these operational limitations of molecular test applied to DTU-specific diagnosis, especially during chronic Chagas disease. Chronic Chagas disease is characterized by a robust antibody response to a range of *T. cruzi* antigens allowing the development of antibody-based tests with high performance for diagnosis purposes. The intrinsic aspects of the immune response repertoire highlight the robustness of these methods to identify single and mixed infection by distinct DTUs. ELISA-based serological methods have been designed for DTU-specific diagnosis of *T. cruzi* infection^25–28^. The combined use of *T. cruzi* peptides and other *T. cruzi* antigens improved the performance of ELISA-based serodiagnosis for Chagas disease. However, these methods showed to be not applicable to all lineage-specific serology. Battacharyya et al.^27^ demonstrated that TSSA lineage serology lacks specificity to detect TcI DTU. More recently, the Chagas-Flow ATE-IgG2a has been presented as an innovative method with outstanding performance for universal and DTU-specific diagnosis of experimental *T. cruzi* infection^29,30^. Moreover, the Human Chagas-Flow ATE-IgG1 methodology has been developed and standardized showing elevated performance for universal diagnosis and post-therapeutic cure assessment in Chagas disease^35^.

The present study aims to upgrade the Human Chagas-Flow ATE-IgG1 and further accomplish the DTU-specific diagnosis of Chagas disease. Three standard *T. cruzi* strains (Colombiana, CL and Y), representative of the major *T. cruzi* DTUs found in Latin America (TcI, TcVI and TcII) were used to setup the Human Chagas-Flow ATE-IgG1 methodology for the universal and DTU-specific diagnosis of Chagas disease.

The results demonstrated that Human Chagas-Flow ATE-IgG1 presented an outstanding performance (Se and Sp = 100%) for universal diagnosis of Chagas disease using pre-selected sets of attributes: “AI 250/40%”, “EVI 250/30%”, “AII 250/40%”, “TII 250/40%” and “EII 250/30%”. These results re-emphasize that TcII DTU has better performance as target antigens for universal diagnosis of Chagas disease. Alessio et al.^29^ have already demonstrated that TcII yielded higher performance indices when used as target antigen for Chagas-Flow ATE-IgG2a applied to the universal diagnosis of experimental *T. cruzi* infection. Corroborating these findings, Bhattacharyya et al.^27^ have shown that without exception all sera from patients with chronic Chagas disease from distinct geographical areas recognized the *T. cruzi* TcII lysate antigen preparation. It is worthy to mention that one of the most widely used antigen for Chagas disease diagnosis is the TcII Y strain^58^. In Brazil, an additional advantage of using TcII antigens for the universal diagnosis of Chagas disease is high prevalence of this *T. cruzi* DTU observed in our country^5,15,40^.

The innovative Human Chagas-Flow ATE-IgG1 methodology also presented a high performance to discriminate the infections with TcI, TcVI or TcII DTUs. The selective use of individual sets of attributes “TI 4,000/50%”, “EI 2,000/50%”, “AVI 8,000/60%” and “TVI 4,000/50%” displayed an overall low or moderate accuracy for DTU-specific diagnosis of Chagas disease. The attribute set “EI 2,000/50%” displayed higher co-positivity (91%) for patients infected with TcI DTU and co-negativity (86% and 88%, respectively) for patients infected with TcVI and TcII DTUs. It has been proposed that TcI DTU is more efficient in inducing over-expression of all anti-*T. cruzi* IgG subclasses as compared to TcII^59^.

Combined strategies, using pre-defined sets of attributes (“TI 4,000/50%”, “EI 2,000/50%”, “AVI 8,000/60%” and “TVI 4,000/50%”) were carried out and decision tree algorithms proposed for two population prototypes (“TcI *vs* TcVI *vs* TcII” and “TcI *vs* TcII”), typifying the distribution of human *T. cruzi* infection in distinct geographic regions. The results demonstrated high performance of combined Human Chagas-Flow ATE-IgG1 for DTU-specific diagnosis of Chagas disease, with outstanding full accuracy (92% and 97%) and cross-validation (LOOCV = 90% and 95%) to discriminate “TcI *vs* TcVI *vs* TcII” and “TcI *vs* TcII” prototypes, respectively. Alessio et al.^29^ analyzing distinct population prototypes in experimental models for *T. cruzi* infection also observed the polarized reactivity of IgG2a in TcI and TcII along with an intermediate distribution pattern for TcVI infected hosts. This phenomenon may reflects the phylogenetic origin of *T. cruzi* DTUs^5^ indicating TcI and TcII as ancestors DTUs with polarized biological features^8,9,60–64^ and TcVI as a hybrid DTU presenting intermediate profile^8,60,61^. Corroborating the previous findings from Alessio et al.^30^ for experimental *T. cruzi* infection, the present work also identified TcI antigen as the root attribute in the decision tree algorithms proposed for DTU-specific diagnosis of human infection.

The present work is a pioneer upgrading of Human Chagas-Flow ATE-IgG1for DTU-specific serotyping of Chagas disease testing a large number of samples from distinct geographical regions. In conclusion, the elevated performance of Human Chagas-Flow ATE-IgG1 qualifies its use for TcI/TcVI/TcII-specific diagnosis of Chagas disease. Additional studies to evaluate the performance of this methodology to diagnose the infection with other *T. cruzi* DTUs are under current investigation. In view of the existence of *T. cruzi* mixed infections in nature and considering the good performance of Chagas-Flow ATE to segregate dual experimental *T. cruzi* infections^30^, other perspective of this work will be to evaluate the reactivity of the serum samples from individuals infected by different genetic groups of the parasite through this methodology. This new method can also be applicable in epidemiological surveys, post-therapeutic monitoring, clinical outcome follow-ups and Chagas disease re-activation in immune-compromised patients.

## Data Availability

All datasets generated for this study are included in the manuscript files.
